# Parapharyngeal and floor‐of‐mouth abscess secondary to tonsillar phlegmon: A rare and unusual cause of Ludwig's angina

**DOI:** 10.1002/ccr3.6325

**Published:** 2022-09-12

**Authors:** Bhavesh V. Tailor, Haran Devakumar, Tharsika Myuran, Dimitrios Ioannidis

**Affiliations:** ^1^ Department of Otolaryngology, Colchester General Hospital East Suffolk and North Essex NHS Foundation Trust Colchester UK

**Keywords:** abscess, Ludwig's angina, parapharyngeal space, peritonsillar abscess, tonsillitis

## Abstract

We present an unusual case of Ludwig's angina secondary to a left tonsillar phlegmon in a previously fit and well 50‐year‐old woman. This tonsillar phlegmon spread along the peritonsillar/parapharyngeal plane to cause a diffuse cellulitis and collection in the submental, sublingual, and submandibular spaces despite empirical intravenous antibiotic therapy.

## Graphical abstract

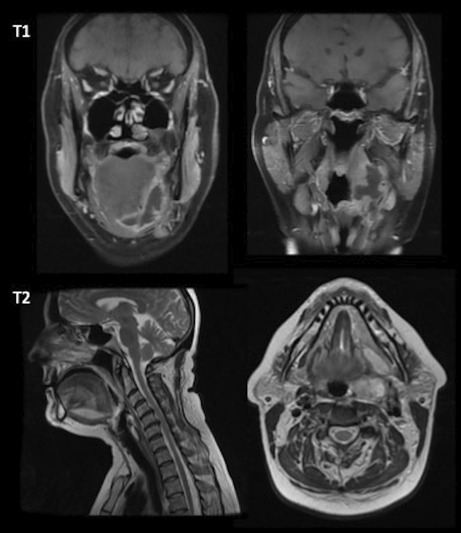

## 1 INTRODUCTION

Peritonsillar infection is a common site of deep neck space infection (DNSI)^1^ and is often described as part of a spectrum of disease from tonsillitis, via peritonsillar cellulitis and phlegmon, culminating in peritonsillar abscess. Such presentations are a frequent source of emergency referrals to acute ENT services.^2^ The mainstay of treatment for peritonsillar infection is intravenous antibiotics and steroids with attempted drainage of peritonsillar abscess if suspected. In cases of untreated or inadequately treated peritonsillar infection, spread of infection may occur through the deep neck tissues, typically to the parapharyngeal or retropharyngeal spaces.^3^

Ludwig's angina is a potentially life‐threatening, diffuse cellulitis of the soft tissues of the floor‐of‐mouth, involving the sublingual, submental, and submandibular spaces. Inflammatory distension of the fascial planes of the neck can progress rapidly, leading to airway obstruction. Effective treatment involves early recognition to secure the airway, intravenous antibiotics and corticosteroids, and consideration of surgical drainage and debridement. Such infections are usually odontogenic in origin.^4^, ^5^

We present an unusual case of Ludwig's angina secondary to a left tonsillar phlegmon. A phlegmon is inflammation of connective tissue, which in this case spread along the peritonsillar/parapharyngeal plane to cause a diffuse cellulitis and collection in the submental, sublingual, and submandibular spaces despite empirical intravenous antibiotic therapy.

## 2 CASE REPORT

A previously fit and well 50‐year‐old woman initially presented to her general practitioner (GP) with a 3‐day history of sore throat and given a course of oral penicillin V for suspected tonsillitis. However, her symptoms progressed rapidly with associated fever and odynophagia, and she presented to the emergency department 48 h later. She had no significant medical or surgical history apart from well‐controlled asthma and denied history of frequent episodes of severe sore throat or tonsillitis and denied dental pain or recent dental extraction. The patient was not diabetic and was not prescribed long‐term corticosteroids or other immunosuppressive medication. Neck examination revealed a firm, non‐fluctuant, exquisitely tender swelling of the floor‐of‐mouth, left submandibular, and left angle of mandible regions, with no associated skin erythema. Examination of the oropharynx was limited by marked trismus and an elevated tongue. Flexible nasoendoscopy revealed swelling of the left pharyngeal wall, with no airway compromise. Blood tests demonstrated neutrophilic leucocytosis and C‐reaction protein level was 343 mg/L.

**FIGURE 1:**
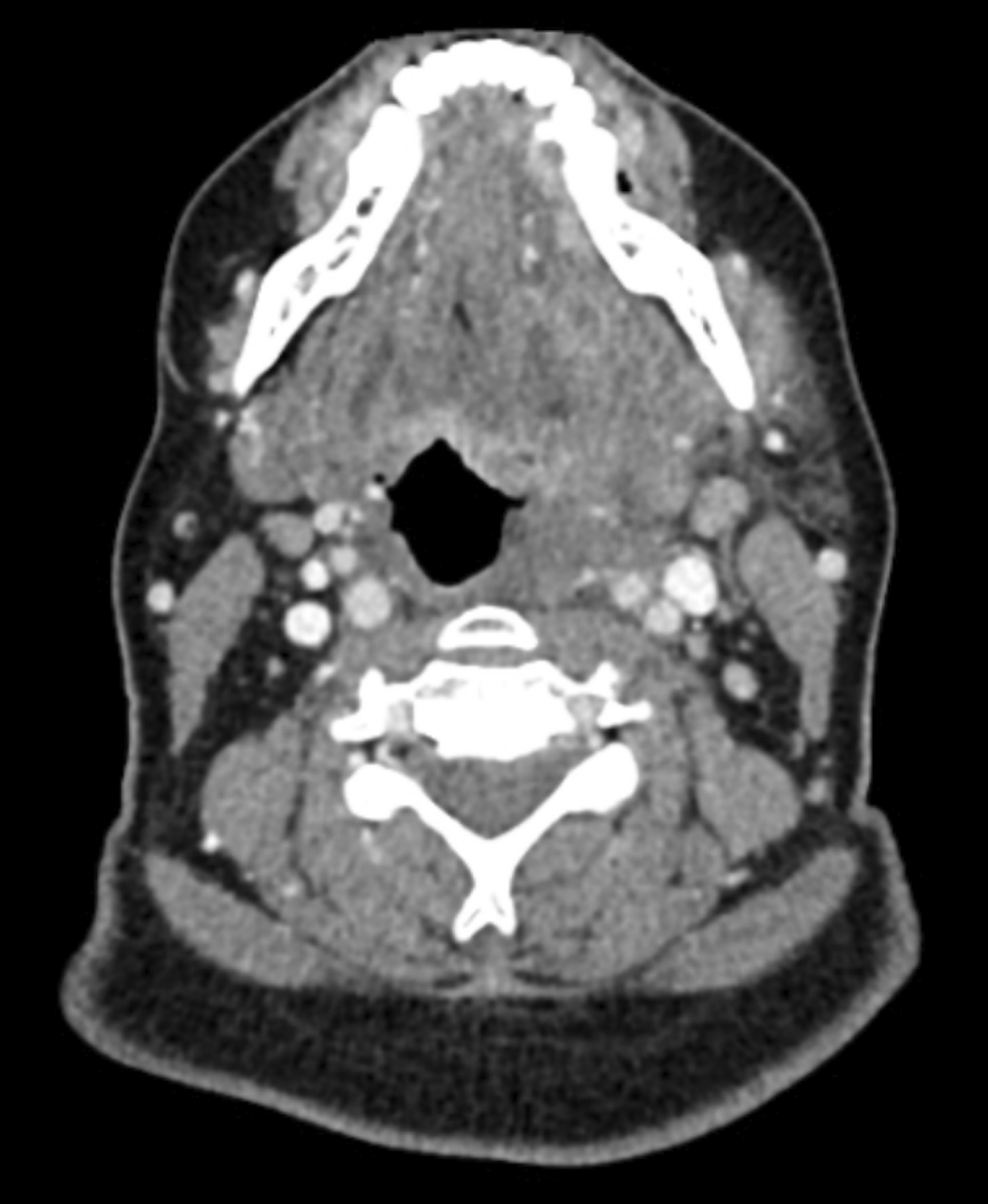
Contrast‐enhanced computed tomography (CT) imaging demonstrating a soft tissue abnormality within the left parapharyngeal space adjacent to the oropharynx but without convincing evidence of enhancement, suggestive of an inflammatory phlegmon.

**FIGURE 2:**
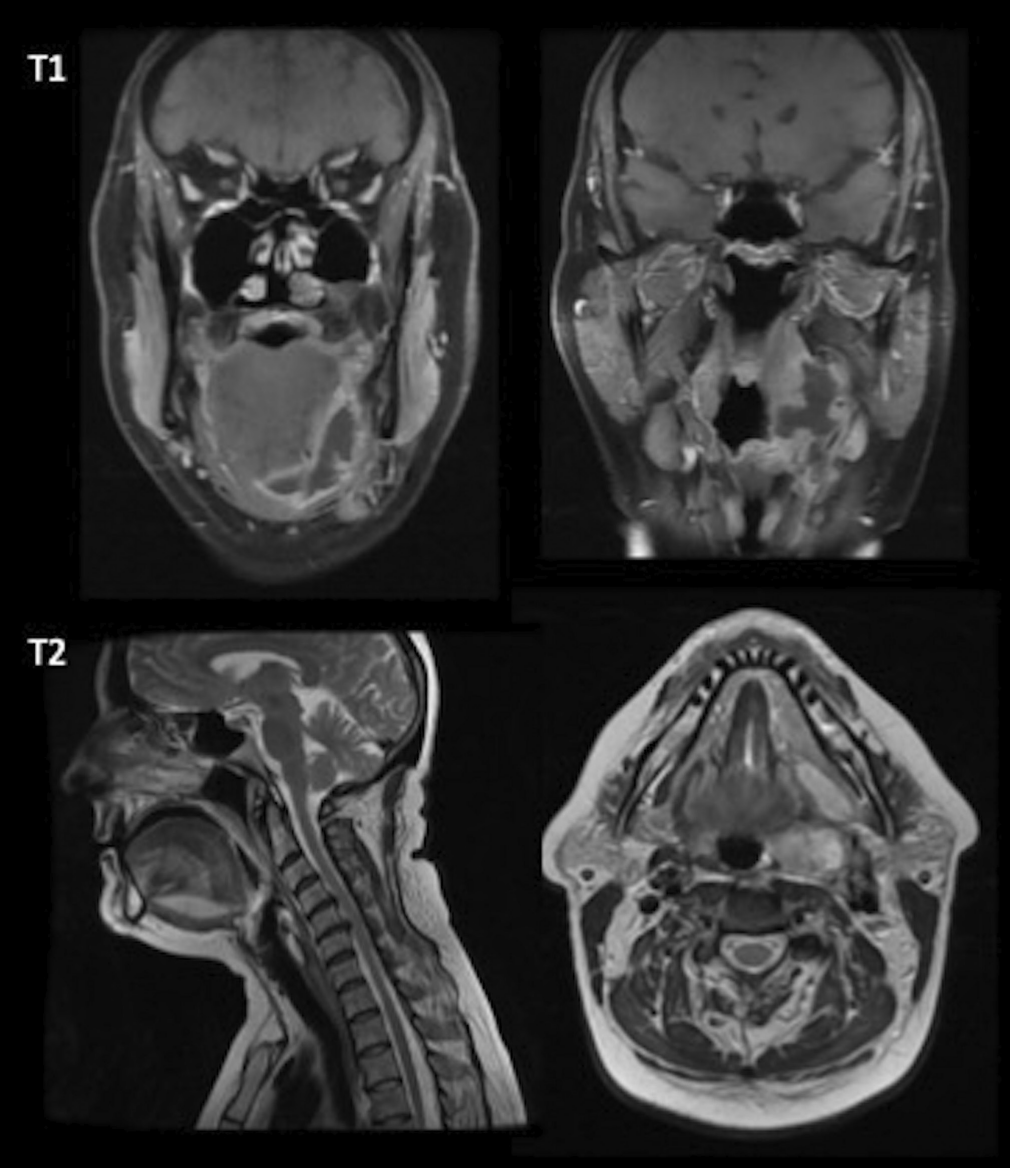
Magnetic resonance imaging (MRI) demonstrating an inflammatory mass lesion and multiloculated collection centered on the left palatine tonsil, which extends to the left lateral and anterior floor‐of‐mouth.

The patient was admitted to hospital and received intravenous antibiotics, intravenous steroids, fluids, and analgesia. She was treated with empiric antibiotic therapy for para−/retropharyngeal abscess (amoxicillin, metronidazole, and gentamicin) as per local hospital protocol. An urgent contrast‐enhanced computed tomography (CT) revealed a soft tissue abnormality within the left parapharyngeal space adjacent to the oropharynx but without convincing evidence of enhancement (Figure 1). A diagnosis of left tonsillar phlegmon was made. Needle aspiration of the left peritonsillar space was attempted but failed to yield any pus. The maxillofacial team was consulted, who deemed an odontogenic cause for the presentation unlikely. A decision was made to continue medical management. Intravenous gentamicin was stopped on Day 3 following advice from the microbiology team. By the morning of Day 4, she was managing small amounts of oral intake and remained afebrile despite the neck swelling and persistent severe pain. Subsequent magnetic resonance imaging (MRI) with contrast demonstrated an inflammatory mass lesion and multiloculated collection centered on the left palatine tonsil, which extended to the left lateral and anterior floor‐of‐mouth, spanning 6.3 cm, in keeping with a Ludwig's angina (Figure 2). There was no evidence of airway compromise. As for antibiotic therapy, intravenous metronidazole was continued while intravenous amoxicillin was switched to intravenous co‐amoxiclav on the advice of the microbiology team. Definitive management entailed hot left tonsillectomy and intraoral abscess incision and drainage. Preoperatively, two discharging areas were identified at the left floor‐of‐mouth and inferolateral aspect of left tonsillar fossa. Both openings were widened intraoperatively to facilitate drainage along with copious irrigation with saline, and a washout revealed they were in communication, avoiding the need for a transcervical drainage approach. A swab was taken, which grew *Streptococcus anginosus*, sensitive to penicillin and erythromycin. Tonsil histology showed ulceration and neutrophilic abscess formation. After 48 h postoperatively, her pain was well‐controlled, she was managing adequate oral intake, and clinical examination demonstrated minimal neck swelling and a healthy tonsillar bed. Following discussion with the microbiology team, she was discharged with a 5‐week course of oral co‐amoxiclav and weaning steroid regime. Upon outpatient review in clinic 6 weeks later, the patient had made a complete recovery, with normal appearances of the floor‐of‐mouth, oropharynx, tongue base and vocal cords on flexible nasoendoscopy.

## 3 DISCUSSION

The peritonsillar space is a potential space between the tonsillar capsule medially and superior pharyngeal constrictor muscle laterally. Due to the presence of loose connective tissue, it is highly susceptible to developing an inflammatory reaction following infection.^6^ Peritonsillar cellulitis or phlegmon is considered a transition phase of the inflammatory process before abscess formation.^7^

Adult DNSI may involve multiple spaces leading to severe complications, and it is sometimes difficult to trace the primary source of the infection.^8^ From the peritonsillar space, infections may extend into the para‐ and/or retropharyngeal spaces.^3^ Klug et al.^9^ reviewed the clinical records of 63 patients with parapharyngeal abscess in a Danish tertiary care center over a 11‐year period and reported that 52% of patients had concomitant peritonsillar abscess. Rarely, from the parapharyngeal space, infections of tonsillar origin may spread into adjacent deep neck spaces such as the submandibular, parotid, and masticator spaces.^10^, ^11^, ^12^, ^13^

A recent narrative systematic review published in 2020 sought to identify the spectrum of complications in patients with peritonsillar abscess and identified only one case of bilateral submandibular cellulitis (and concurrent parapharyngeal abscess) among 334 published cases of peritonsillar abscess with associated complications in the literature.^3^, ^12^ The 75‐year‐old gentleman refused hot tonsillectomy and subsequently developed submandibular space abscess, necessitating abscess drainage via a cervical approach.^12^ Matsuura describes a case of a 68‐year‐old gentleman with ulcerative colitis who presented with peritonsillar abscess complicated by Ludwig's angina. The patient initially refused needle aspiration and developed submandibular erythema with swelling 5 days later, requiring submandibular incision and drainage as well as a 6‐week course of intravenous and oral antibiotics.^13^

In the present case, because initial CT imaging of the neck demonstrated a non‐enhancing soft tissue abnormality within the parapharyngeal space and attempted needle aspiration of the peritonsillar space did not yield pus, a diagnosis of tonsillar phlegmon was made. Antibiotic therapy was empirically initiated following microbiology guidelines based on local epidemiology. The rationale for multiple broad‐spectrum antimicrobial therapy in DNSI is to ensure adequate cover for all likely pathogens, both aerobic and anaerobic bacteria originating from the pharyngeal flora.^14^ Our hospital protocol recommended intravenous amoxicillin, metronidazole, and gentamicin, although various antibiotic regimes exist depending on geographical region. Gram‐positive cocci, such as streptococci and staphylococci, are the most common bacterial species isolated.^14^, ^15^

However, despite aggressive medical management with intravenous antibiotics and steroids, the patient subsequently developed a left parapharyngeal and floor‐of‐mouth abscess. It is possible that a small abscess was present during the early phases of the patient's illness, but unfortunately too subtle to detect on CT. The prolonged use of antibiotics without surgical drainage could have created an antibioma (i.e., sterile abscess) due to localization of pus, often reported in odontogenic infections.^16^ This might explain why the patient did not obtain significant relief of symptoms by Day 4 of admission. Although *Streptococcus anginosus* was subsequently isolated from the swab taken intraoperatively, this may have represented contamination from oral commensal bacteria.

Unusually, the patient did not present with risk factors typically associated with spread of DNSI, such as older age, diabetes mellitus, immunosuppression, or underlying systemic disease.^17^, ^18^ There is no standard surgical protocol for managing peritonsillitis with involvement of adjacent deep neck spaces, although hot (or abscess) tonsillectomy and intraoral incision and drainage, as performed in this case, is recommended to reduce risk of further abscess extension.^9^, ^12^

Although there are frequent reports of peritonsillar abscess complicated by parapharyngeal extension, the present case is unique in that initial imaging demonstrated a tonsillar phlegmon with some mass effect but no convincing evidence of collection. This tonsillar phlegmon spread along the peritonsillar/parapharyngeal plane to cause a diffuse cellulitis and collection in the floor‐of‐mouth despite intravenous antibiotic therapy. Although Ludwig's angina is usually odontogenic in origin, it is important to highlight that other etiologies are possible, although less common. Throughout the course of admission, there was no evidence of airway compromise. However, following 4 days of intravenous antibiotics and steroids, the neck swelling showed little improvement, which, in retrospect, likely reflected the evolving clinical presentation from phlegmon to abscess. Serial imaging should always be considered if the initial response to medical management is unsatisfactory.

## 4 CONCLUSION

Peritonsillar cellulitis or phlegmon is considered a transition phase of the inflammatory process before abscess formation. In cases of untreated or inadequately treated peritonsillar infection, spread of infection may occur through the deep neck tissues. This case of left tonsillar phlegmon spread along the peritonsillar/parapharyngeal plane to cause a diffuse cellulitis and collection in the floor‐of‐mouth despite empirical intravenous antibiotic therapy. Although Ludwig's angina is usually odontogenic in origin, it is important to highlight that other etiologies are possible, although less common.

## AUTHOR CONTRIBUTIONS

BVT collected the data for the case report, performed the literature search, and wrote the original manuscript. HD, TM, and DI contributed to the literature search and revised the manuscript for its scientific basis. All authors read and approved the final manuscript.

## CONFLICT OF INTEREST

The authors declare that they have no competing or conflict of interests.

## CONSENT

Written informed consent was obtained from the patient for the publication of this case report and any accompanying images.

## Data Availability

The data that support the findings of this study are available from the corresponding author upon reasonable request.
